# Energy Harvesting Based Body Area Networks for Smart Health

**DOI:** 10.3390/s17071602

**Published:** 2017-07-10

**Authors:** Yixue Hao, Limei Peng, Huimin Lu, Mohammad Mehedi Hassan, Atif Alamri

**Affiliations:** 1 School of Computer Science and Technology, Huazhong University of Science and Technology, Wuhan 430074, China; yixuehao@hust.edu.cn; 2 Department of Industrial and Information System Engineering, Ajou University, Suwon 443749 , Korea; 3 Department of Mechanical and Control Engineering, Kyushu Institute of Technology, Fukuoka prefecture 8048550, Japan; luhuimin@ieee.org; 4 College of Computer and Information Sciences, King Saud University, Riyadh 11543, Saudi Arabia; mmhassan@ksu.edu.sa (M.M.H.); atif@ksu.edu.sa (A.A.)

**Keywords:** energy harvesting, wireless powered body area network, resource allocation

## Abstract

Body area networks (BANs) are configured with a great number of ultra-low power consumption wearable devices, which constantly monitor physiological signals of the human body and thus realize intelligent monitoring. However, the collection and transfer of human body signals consume energy, and considering the comfort demand of wearable devices, both the size and the capacity of a wearable device’s battery are limited. Thus, minimizing the energy consumption of wearable devices and optimizing the BAN energy efficiency is still a challenging problem. Therefore, in this paper, we propose an energy harvesting-based BAN for smart health and discuss an optimal resource allocation scheme to improve BAN energy efficiency. Specifically, firstly, considering energy harvesting in a BAN and the time limits of human body signal transfer, we formulate the energy efficiency optimization problem of time division for wireless energy transfer and wireless information transfer. Secondly, we convert the optimization problem into a convex optimization problem under a linear constraint and propose a closed-form solution to the problem. Finally, simulation results proved that when the size of data acquired by the wearable devices is small, the proportion of energy consumed by the circuit and signal acquisition of the wearable devices is big, and when the size of data acquired by the wearable devices is big, the energy consumed by the signal transfer of the wearable device is decisive.

## 1. Introduction

Body area networks (BANs) are small wireless sensor networks (WSNs) which support a lot of medical applications and provide a solution for smart health monitoring [1]. For an exhaustive introduction to BANs, we refer the reader to [2,3]. BANs are configured with ultra-low power consumption wearable devices [4] and medical sensors [5] (such as digestible medical electronics). These sensors constantly monitor physiological signals and movement data of the human body; they transfer such signals and data to the cloud for analysis, thus realizing intelligent monitoring of the user’s health [6,7].

However, data collection and transfer of BAN sensors consume energy. To increase the comfort of wearable devices, the battery of wearable devices is usually small, thus the battery’s capacity is limited [8,9]. Old batteries need to be replaced frequently or recharged regularly. For medical sensors (such as digestible sensors), it is impossible to replace or recharge the battery. Thus, minimizing the energy consumption of wearable devices and optimizing BAN energy efficiency is still a challenging problem [10].

Nowadays, most research concerning BAN energy efficiency optimization focuses on the design of the routing algorithm [11,12], duty-cycle-based data collation [1], data reduction and compressed sending [13,14], and cross-layer design [15]. For example, some papers have proposed novel approaches to reduce energy consumption through an adaptive routing algorithm [16], dynamic programming for heterogeneous networks [17], and voltage/frequency scaling [18], and secure data transmission [19]. Other papers have dealt with the issue of data-generation uncertainty in the optimal design of BANs: [20,21] proposed a robust optimization model solved by fast mixed integer programming heuristics, based on the algorithm for robust capacitated network design proposed in [22]; Reference [23] has instead investigated the adoption of a min–max regret model. However, few works realize BAN energy efficiency optimization through energy harvesting. Generally, energy in the BAN sensor can be harvested in the following three ways:

- Energy harvesting through the environment: some sensors harvest energy through renewable energy sources (including solar, wind, and luminous energy resources).
- Energy harvesting through the human body: some sensors harvest energy from their own heat energy, bio-energy, body surface friction and body movement.
- Energy harvesting through a wireless signal: some sensors harvest energy by acquiring wireless signals.

For energy harvesting through the environment, it is impossible for the user to be exposed to strong sunshine or strong wind for long time and this energy harvesting mode depends greatly on the weather and other conditions, which may lead to a longer delay [24,25]. Thus, it is not applicable to BANs. For energy harvesting through the human body, the bio-energy of the human body is unstable and may result in unreliable energy production; furthermore, wearing an additional energy-harvesting device may result in discomfort [26]. Thus, it is also not applicable to a BAN. For energy harvesting through a wireless signal or radio frequency (RF) energy harvesting [27,28], considering that wireless signals exist everywhere constantly and controllably, it is a feasible approach to provide reliable energy to the low power consumption sensors.

A RF energy harvesting based BAN includes two stages: wireless energy transfer (WET) and wireless information transfer (WIT) [29,30]. A challenging problem of the simultaneous wireless information and power transfer (SWIPT) is how to allocate resources between the WET and WIT, so as to minimize the energy consumed by the network. Some research has included primary exploration and discussion on BANs. For example, Abubaka et al. [5] proved in their research that WET to the sensor in the digestive tract could be realized through an antenna outside the human body and such energy was sufficient to keep the sensor working normally, including being able to monitor the environment and temperature of the digestive tract and the special nutrition cost.However, these studies failed to take into consideration the energy consumed by the circuit and data collection and processing of the sensor. In fact, the small size of the sensor in a BAN may result in a great proportion of energy being consumed by the circuit, data collection and processing. Thus, it is obviously impossible to neglect such power consumption.

In this paper, we propose the energy harvesting-based BAN, i.e., the sensor in a BAN can harvest energy from access points and transmit the collected data to access points. Furthermore, we study the resources allocation scheme that realizes minimized energy consumption in a BAN. To be specific, the main results and contributions of this paper include the following:

- We introduce energy harvesting into a BAN to improve BAN energy efficiency. Compared with traditional BANs, the energy harvesting based BAN proposed in this paper can significantly improve the BAN energy efficiency.
- We formulate the optimization problem concerning time allocation for the WET and WIT in a BAN, with the aim of minimizing energy consumption in the sensor when considering the WIT and WET time limits. Furthermore, we convert such a problem into a convex optimization problem under linear constraints.
- We propose a closed-form solution to the optimization problem based on Karush–Kuhn–Tucker (KKT) conditions. Simulation results showed that when the size of data acquired by the wearable devices is small, the proportion of energy consumed by the circuit and information collection of the wearable devices is big, and when the size of data acquired by the wearable devices is big, the energy consumed by information transfer of the wearable device is decisive.

The remainder of this article is organized as follows. The system model is described in Section 2. We first formulate an optimization problem to minimize the energy consumption of the energy harvesting-based BAN. Then, we transform the problem to a convex optimization problem with linear constraints and propose a closed-form solution in Section 3. Our experimental results and discussions are provided in Section 4. Finally, Section 5 concludes this paper.

## 2. System Model

In this section, we introduce the network architecture of an energy harvesting-based BAN, as well as the WET and WIT model based on the time division multiple access (TDMA) protocol.

### 2.1. Energy Harvesting-Based Body Area Networks Model

**Figure 1:**
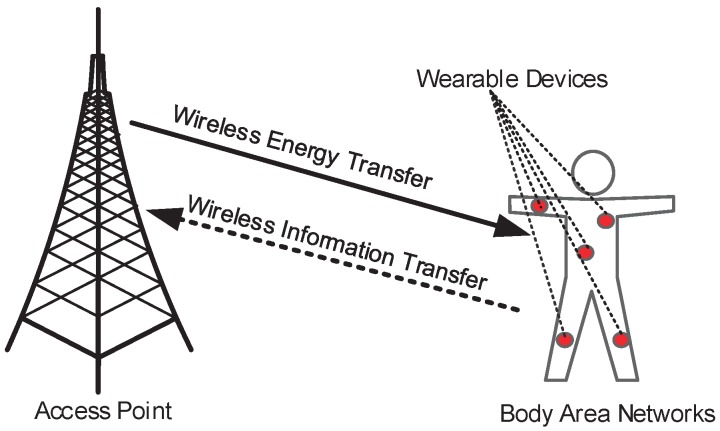
Energy harvesting-based body area network.

We consider an energy harvesting BAN as shown in Figure 1; the ultra-low power consumption sensor configured to the BAN could collect the physiological signals of the human body, such as electrocardiography signals. The access point recharged the sensor through WET at a fixed interval and the sensor needed to deliver collected physiological signals of the human body to the access point. That is, the human body sensor harvested energy from the access point. When the sensor acquired energy, some of the harvested energy was used for signal transfer, while some of the harvested energy was consumed by the circuit and data acquisition of the sensor. In this paper, we assume that the access point has a stable energy supply and can provide sufficient energy to the sensor.

**Figure 2:**
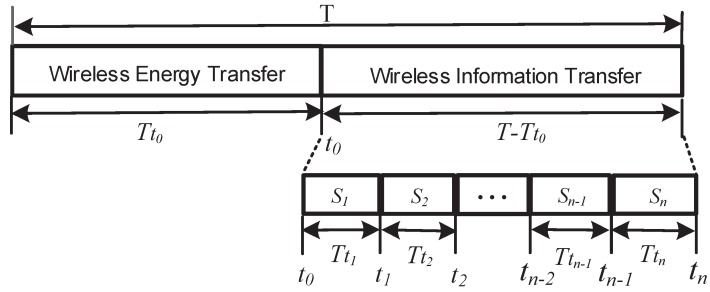
The time division multiple access (TDMA)-based transmission protocol.

In this paper, the transfer protocol used is the TDMA protocol as shown in Figure 2. We assume that there are *n* sensors within the area covered by the access point and these sensors are denoted as $S=\{S_{1},S_{2},\cdots ,S_{n}\}$. Let $t_{0},t_{1},\cdots ,t_{n}$ denote the time slot and $T_{t_{0}},T_{t_{1}},\cdots ,T_{t_{n}}$ represent the duration of the time slot. Considering that the WET and WIT will be finished within the period *T*, *T* can be divided into n+1 duration of the time slot, i.e.,

(1) $$\sum_{i=0}^{n}T_{t_{i}}=T.$$

In the duration of the time slot $t_{0}$, WET is performed and the access point transfers energy to *n* sensors by way of signal broadcasting. In the duration of the time slot $t_{1},t_{2},\cdots ,t_{n}$, *n* sensors perform WIT and transfer information to the access point through the harvested energy.

### 2.2. Transmission Model

The transmission modes included WET and WIT. In WET, the sensor harvests energy through the access point. In WET, the sensor transfers acquired information to the access point. Detailed models are shown below.

*Wireless energy transfer model:* Let $P_{b}$ denote the access point transmission power; then, according to the the work of You et al. [31], the energy harvested by the sensor $S_{i}$, denoted as $E_{i}^{H}$, is given as:

(2) $$E_{i}^{H}=\eta P_{b}h_{i}^{DL}T_{t_{0}}$$

where $h_{i}^{DL}$ is the channel gains from the access point to the sensor $S_{i}$, and η (0<η<1) is the energy converting efficiency.

*Information transfer model:* After the sensor harvested energy, it is necessary to transmit the collected information to the access point. The channel gain of the sensor $S_{i}$ is defined as $h_{i}$, and the transfer power of $S_{i}$ is defined as $p_{i}$. Then, according to the work of You et al. [31], the transfer rate of $S_{i}$ in the time slot $t_{i}$, denoted as $r_{i}$, is given as:

(3) $$r_{i}=B\log_{2}\left(1+\frac{p_{i}h_{i}}{\sigma^{2}}\right)$$

where $\sigma^{2}$ is the variance of complex white Gaussian noise, *B* is the channel bandwidth from the sensor $S_{i}$ to the access point.

### 2.3. Energy Consumption Model

The energy consumption of the sensor $S_{i}$ in $T_{t_{i}}$ can be divided into the following three parts:

1. Energy consumed by the circuit of the sensor $S_{i}$. Considering that the sensor works constantly, this energy is constant and we denote it as $E_{i}^{c}$.
2. Energy consumed by signals (such as the perception, collection and storage of signals) processed by the sensor $S_{i}$. This part of energy consumption is associated with the data size which is denoted as $\omega_{i}$ to be processed. Let $\gamma_{i}$ denote the energy consumed by the processing of one bit of data. Then, this part of energy can be expressed as $E_{i}^{proc}=\gamma_{i}\omega_{i}$.
3. Energy consumed for information transfer by the sensor $S_{i}$. This part of energy consumption is also related to the data size $\omega_{i}$ and can be represented as $E_{i}^{tran}=p_{i}\omega_{i}/r_{i}$.

According to the work of You et al. [31], under a given time constraint, the most energy-efficient data transfer policy is fixed-rate transmission over the whole time slot. Thus, for the sensor $S_{i}$ in the duration of time slot $t_{i}$, the lowest energy consumption transfer rate is fixed at $r_{i}=\omega_{i}/T_{t_{i}}$. Based on the above discussion and Equation (5), we can rewrite the $E_{i}^{tran}$ as follows:

(4) $$E_{i}^{tran}=p_{i}T_{t_{i}}=\left(2^{\frac{\omega_{i}}{BT_{t_{i}}}}-1\right)\frac{\sigma^{2}T_{t_{i}}}{h_{i}}$$

Thus, the total energy consumed by the sensor $S_{i}$, denoted as $E_{i}^{loc}$, can be obtained as follows:

(5) $$E_{i}^{loc}=E_{i}^{c}+E_{i}^{proc}+E_{i}^{tran}=E_{i}^{c}+\gamma_{i}\omega_{i}+\left(2^{\frac{\omega_{i}}{Bt_{i}}}-1\right)\frac{\sigma^{2}t_{i}}{h_{i}}$$

## 3. Problem Formulation and Solution

In this section, we introduce the optimization problem of BAN time allocation, with the aim of achieving rational allocation of resources, thus minimizing the energy consumption of the sensor.

### 3.1. Problem Formulation

In this paper, we assume that $T_{t_{0}}$ is fixed. That is, the system gives the parameter $T_{t_{0}}$ earlier, in which energy is transferred to sensors in the human body. $T_{\mathrm{opt}}=\left[T_{t_{1}},T_{t_{2}},\cdots ,T_{t_{n}}\right]$ with the aim of dividing the period $T-T_{t_{0}}$ rationally, thus minimizing the energy consumption of the sensor. In view of the discussion described in Section 2, the following optimization problems can be obtained:

(6) $$\begin{array}{cc} P1:\underset{T_{t_{i}}}{\mathrm{minimize}}\; & \sum_{i=1}^{n}\left[E_{i}^{c}+\gamma_{i}\omega_{i}+\left(2^{\frac{\omega_{i}}{BT_{t_{i}}}}-1\right)\frac{\sigma^{2}T_{t_{i}}}{h_{i}}\right] \end{array}$$

(7) $$\begin{array}{cc} \mathrm{subject}\;\mathrm{to}:\;\;\; & \sum_{i=1}^{n}T_{t_{i}}\leq T-T_{t_{0}},\;T_{t_{i}}\geq 0,\;i=1,2,\cdots ,n. \end{array}$$

(8) $$\begin{array}{c} E_{i}^{c}+\gamma_{i}\omega_{i}+\left(2^{\frac{\omega_{i}}{BT_{t_{i}}}}-1\right)\frac{\sigma^{2}T_{t_{i}}}{h_{i}}\leq \eta_{i}P_{b}h_{i}^{DL}T_{t_{0}},\;i=1,2,\cdots ,n. \end{array}$$

where the objective function (6) is the energy consumption of the minimized sensor. The constraint condition (7) meant that the WIT of *n* sensors was finished within the time duration $T-T_{t_{0}}$.The constraint condition (8) meant that the energy consumed by the sensor should not exceed the harvested energy.

To solve the above optimization problem, we further adapt the problem and combine the constraint condition (8) and the objective function (6); then, we can obtain the energy savings $\Delta E_{i}$ as follows:

(9) $$\Delta E_{i}=\eta_{i}P_{b}h_{i}^{DL}T_{t_{0}}-\left[E_{i}^{c}+\gamma_{i}\omega_{i}+\left(2^{\frac{\omega_{i}}{BT_{t_{i}}}}-1\right)\frac{\sigma^{2}T_{t_{i}}}{h_{i}}\right]$$

Thus, the following optimization problem, which is equivalent to P1, can be obtained:

(10) $$\begin{array}{cc} P2:\underset{t_{i}}{\mathrm{minimize}}\; & -\sum_{i=1}^{n}\Delta E_{i} \end{array}$$

(11) $$\begin{array}{cc} \mathrm{subject}\;\mathrm{to}:\; & \sum_{i=1}^{n}t_{i}\leq T-T_{t_{0}},\;T_{t_{i}}\geq 0,\;i=1,2,\cdots ,n. \end{array}$$

In the next subsection, we characterize the solution of the problem.

### 3.2. Closed-Form Solution

As for the optimization problem described above, it can be proven to be a convex optimization problem as follows.

**Theorem** **1.** *Problem P2 is the convex optimization problem*.

**Proof** **of** **Theorem** **1.** Define the function $f\left(x\right)=2^{\frac{x}{B}}-1$; then, we can obtain the first and the second derivative of f(x) as follows:

$$f^{\prime }\left(x\right)=\frac{\ln2}{B}2^{\frac{x}{B}},\;f^{\prime \prime }\left(x\right)={\left(\frac{\ln2}{B}\right)}^{2}2^{\frac{x}{B}}$$

Here, it is clear that $f^{\prime \prime }\left(x\right)>0$, thus f(x) is convex. Considering the perspective function of f(x), g(x,t)=tf(x/t) is also convex with respect to (t,x). It is obvious that $E_{i}^{t}=\frac{t_{i}}{h_{k}^{2}}\left(2^{\frac{\omega_{i}/t_{i}}{B}}-1\right)\sigma^{2}$ is the convex function of $t_{i}$. Therefore, $E_{i}^{c}+E_{i}^{proc}+E_{i}^{tran}$ is a convex function. Since the sum of convex functions is still convex, the objective function is a convex function. The restriction is a linear constraint. Thus, the optimization problem is convex [32]. ☐

As for problem P2, we can define its Lagrange function as follows:

(12) $$L=\sum_{i=1}^{n}\left[E_{i}^{c}+\gamma_{i}\omega_{i}+\left(2^{\frac{\omega_{i}}{BT_{t_{i}}}}-1\right)\frac{\sigma^{2}T_{t_{i}}}{h_{i}}-\eta_{i}P_{b}h_{i}^{DL}T_{t_{0}}\right]+\lambda \left(\sum_{i=1}^{n}T_{t_{i}}+T_{t_{0}}-T\right)$$

where λ is the Lagrange multiplier. It is assumed that $T_{t_{i}}^{*}$ and $\lambda^{*}$ are the optimal solutions of problem P2 and its dual problem, respectively. Based on the Karush–Kuhn–Tucker (KTT) conditions, the following conditions can be obtained:

(13) $$\begin{array}{c} \sum_{i=1}^{n}T_{t_{i}}\leq T-T_{t_{0}} \end{array}$$

(14) $$\begin{array}{c} \lambda^{*}\geq 0 \end{array}$$

(15) $$\begin{array}{c} \lambda^{*}\left(\sum_{i=1}^{n}T_{t_{i}}^{*}+T_{t_{0}}-T\right)=0 \end{array}$$

(16) $$\begin{array}{c} \frac{\partial L}{\partial T_{t_{i}}^{*}}=2^{\frac{\omega_{i}}{BT_{t_{i}}}}\frac{\sigma^{2}}{h_{i}}\left(1-\frac{\ln2\omega_{i}}{BT_{t_{i}}}\right)-\frac{\sigma^{2}}{h_{i}}+\lambda^{*}=0 \end{array}$$

Based on these conditions, the optimal time allocation scheme can be derived as the following theorem.

**Theorem** **2.** *The optimal time allocation scheme for Problem P2 is shown as follows*.

(17) $$T_{t_{i}}^{*}=\frac{\ln2\omega_{i}}{B(1+W\left(\frac{h_{i}\lambda^{*}-\sigma^{2}}{e\sigma^{2}}\right))}$$

where W(x) is the Lambert function and $\sum_{i=1}^{n}T_{t_{i}}^{*}=T-T_{t_{0}}$.

**Proof** **of** **Theorem** **2.** For the sake of simplicity, we define $A=\frac{\sigma^{2}}{h_{i}}$, $x=\frac{\omega_{i}}{BT_{t_{i}}^{*}}$. Based on (16), it has:

(18) $$2^{x}\left(1-x\ln2\right)=\frac{A-\lambda^{*}}{A}$$

Using the properties of exponential functions, we can obtain:

(19) $$e^{-x\ln2}=\frac{A\ln2}{\lambda^{*}-A}\left(x-\frac{1}{\ln2}\right)$$

Based on the definition of the Lambert function, the solution of (19) can be obtained:

(20) $$x=\frac{1+W(\frac{h_{i}\lambda^{*}-\sigma^{2}}{e\sigma^{2}})}{\ln2}$$

Thus, the optimal resource allocation scheme will be given as follows:

(21) $$T_{t_{i}}^{*}=\frac{\ln2\omega_{i}}{B(1+W\left(\frac{h_{i}\lambda^{*}-\sigma^{2}}{e\sigma^{2}}\right))}$$

Furthermore, based on (15), we can obtain $\lambda^{*}=0$ or $\sum_{i=1}^{n}T_{t_{i}}^{*}+T_{t_{0}}-T=0$.

(1) If $\lambda^{*}=0$, the optimal resource allocation scheme would be shown as below:

(22) $$T_{t_{i}}^{*}=\frac{\ln2\omega_{i}}{B(1+W\left(-e^{-1}\right))}$$

since $W(-e^{-1})=-1$, the denominator of (22) is zero. Thus, $\lambda^{*}$ satisfy $\lambda^{*}>0$.

(2) If $\lambda^{*}>0$, the optimal resource allocation scheme would be obtained as follows:

(23) $$T_{t_{i}}^{*}=\frac{\ln2\omega_{i}}{B(1+W\left(\frac{h_{i}\lambda^{*}-\sigma^{2}}{e\sigma^{2}}\right))}$$

and it satisfies $\sum_{i=1}^{n}T_{t_{i}}^{*}=T-T_{t_{0}}$. ☐

## 4. Simulation Results

In this section, we conduct the simulation experiments. Firstly, we set the simulation parameter, then we evaluate the performance.

### 4.1. Parameter Setting

We assume that there are 6 sensors in BAN. As for the WET stage, assume that T=1 s and the WET time slot is $t_{0}=200$ ms. The channels $h_{i}$ are modeled as independent Rayleigh fading with average power loss set as $10^{-3}$. We set the transmitted power of the access point as $P_{b}=100$ W. For the WIT stage, we set the bandwidth as W=5 MHz and the variance of complex white Gaussian channel noise as $\sigma =10^{-9}$ W. In this paper, for the purpose of convenience, we assume that the sensors have equal circuit consumption $E_{i}^{c}$ and energy $\gamma_{i}$ consumed for processing one bit of data. Hence, in this paper, $E_{i}=0.001$ J and $\gamma =10^{-4}$ J/bit. The data size to be transferred followed uniform distribution with the mean value ω=1000 bit.

### 4.2. Energy Cost of Sensors

We give the effects of data size ω on the energy consumption of the sensor. The *X*-axis represents the size of data (Kbits), the *Y*-axis is the energy consumption of all the sensors and we used logarithmic coordinates to the axis *Y*, and the corresponding unit of measurement is $\log_{10}$ Joule.

**Figure 3:**
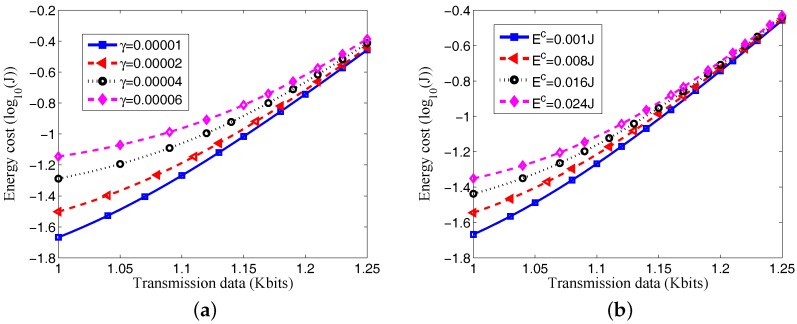
(**a**) Effects of transmission data size ω on energy consumption under different γ; (**b**) Effects of transmission data size ω on energy consumption under different $E_{c}$.

**Figure 4:**
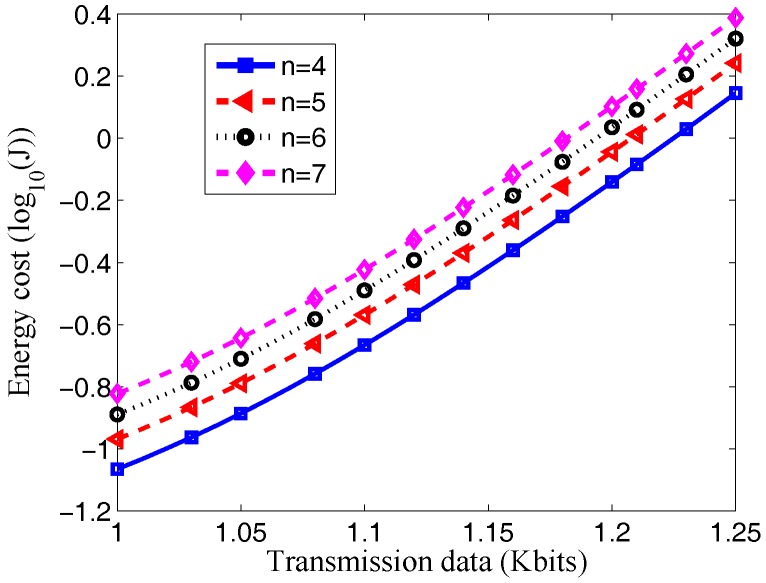
Effects of transmission data size ω on energy consumption under different *n*.

It can be seen from Figure 3 that along with the increase in data size ω, more energy will be consumed. It is because the increase of data size requires more energy for data transfer and information processing. It can be observed from Figure 3a that with the same size of data transfer, more energy consumed in processing each bit of data suggests more energy consumed by the system. It is clear in Figure 3b that with the same transferred data size, the bigger the energy $E^{c}$ consumed by the circuit of the sensor, the bigger the energy consumed by the sensor. Furthermore, it can be deduced based on these two figures that when the transfer data size is smaller, for instance, ω=1000 bits, the difference between curves is bigger. It is because when ω is smaller, the energy consumed by the circuit of the sensor and the energy consumed by information processing take a greater proportion; when the transfer data size is bigger, such as ω=1200 bits, the difference between curves decreases because when the transfer data size ω is big, the energy consumption of the sensor is mainly energy consumed for data transfer. Moreover, Figure 4 shows that more energy is required as the number of sensors *n* increases. The reason is that as the number of sensors increases, sensors can collect more data and deliver the data to the access point, which consumes more energy.

### 4.3. Time Duration Allocation of Sensors

**Figure 5:**
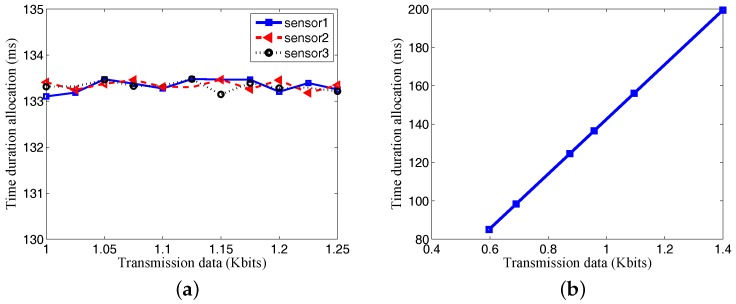
(**a**) Effects of transmission data size ω on time duration allocation under different sensors; (**b**) Effects of transmission data size ω on time duration allocation under a given data size.

In this subsection, we discuss the relationship between transmission data size ω and time duration allocation $T_{t_{i}}$. Figure 5a shows the effects of transmission data size ω on the time duration allocation. The *X*-axis represents the mean value of transmission data size that follows a uniform distribution. The *Y*-axis is the time duration allocation. From the figure, we can observe that the time duration allocated by sensors 1, 2 and 3 varies little with the increment of transmission data size. Compared with Figure 3, we can conclude that the size of data transmitted has a greater impact on energy cost than on the time duration allocation. Figure 5b shows how the system allocates time duration for each sensor when given a set of transmission data sizes. The *X*-axis represents the specific transmission data size and the *Y*-axis indicates the time duration allocation. From the figure, we can observe that the time duration allocation increases with the increment of given transmission data.

## 5. Conclusions

In this paper, we proposed an energy harvesting-based body sensor network, and a method based on time division multiple access (TDMA); we built upon the optimization problem of time division for wireless information transfer and proved that this optimization problem was actually a convex optimization problem; we gave a closed-form solution to the problem. The simulation results of the experiment indicated that when the size of data acquired by the sensor was small, the energy consumption of the sensor was mainly energy consumed by the sensor circuit and energy consumed for data acquisition; when the size of data acquired by the sensor was big, the energy consumed by the sensor for data transfer was decisive.
